# Mental health among children and adolescents: Construct validity, reliability, and parent-adolescent agreement on the ‘Strengths and Difficulties Questionnaire’ in Chile

**DOI:** 10.1371/journal.pone.0191809

**Published:** 2018-02-05

**Authors:** Jorge Gaete, Jesus Montero-Marin, Daniela Valenzuela, Cristian A. Rojas-Barahona, Esterbina Olivares, Ricardo Araya

**Affiliations:** 1 Department of Public Health and Epidemiology, Faculty of Medicine, Universidad de los Andes, Santiago, Chile; 2 Department of Population Health, London School of Hygiene and Tropical Medicine, London, United Kingdom; 3 Primary Care Prevention and Health Promotion Research Network (RedIAPP), Centro de Investigación Biomédica en Red de Salud Mental, CIBERSAM, Zaragoza, Spain; 4 School of Psychology, Universidad de los Andes, Santiago, Chile; 5 Faculty of Education, Pontificia Universidad Católica de Chile, Santiago, Chile; 6 School of Nursing (Campus San Felipe), Universidad de Valparaíso, San Felipe, Chile; 7 Centre for Global Mental Health and Primary Care Research, Health Service and Population Research Department, Institute of Psychiatry, Psychology, and Neuroscience, King’s College London, London, United Kingdom; IRCCS E. Medea, ITALY

## Abstract

The Strengths and Difficulties Questionnaire (SDQ) is a screening tool used to measure psychological functioning among children and adolescents. It has been extensively used worldwide, but its psychometric properties, such as internal structure and reliability, seem to vary across countries. This is the first study exploring the construct validity and reliability of the Spanish version of SDQ among early adolescents (self-reported) and their parents in Latin America. A total of 1,284 early adolescents (9–15 years) and their parents answered the SDQ. We also collected demographic variables. A confirmatory factor analysis was conducted to assess the latent structure of the SDQ. We also used the multitrait-multimethod analysis to separate the true variance on the constructs from variance resulting from measurement methods (self-report vs. parent report), and evaluated the agreement between adolescents and their parents. We found that the original five-factor model was a good solution and the resulting sub-scales had good internal consistency. We also found that the self-reported and parental versions of SDQ provide different information, which are complementary and provide a better picture of the emotional, social, and conduct problems of adolescents. We have added evidence for the construct validity and reliability of the Spanish self-reported and parental SDQ versions in a Chilean sample.

## Introduction

Mental health is an important problem worldwide, and Chile is no exception. In general, the main cause of years lost because of disability among the population aged 10–24 years old is neuropsychiatric disorder (45%) [1]. Mental health problems have an important effect, not only on the lives of the affected individuals, but also on the people around them (family, school, peers). Most of the psychiatric problems we see in adult life start during childhood or adolescence [2]. Recent reports show that the prevalence of psychiatric disorders among Chilean adolescents reaches 16.5%–18.2% [3, 4], but only a minority of affected adolescents make contact with the specialized services they require.

Detecting mental health problems early increases the chances of reduced burden and future complications [5]. In recent years, interest in developing instruments to detect these emotional and behavioural disorders has been increasing. Moreover, the World Health Organization has repetitively advocated using screening tools in primary care settings and schools [6–8]. These instruments should be valid and reliable for assessing mental health among adolescents, but should also be short, simple, and culturally adapted/tested.

The most widely used screening tests for detecting psychological and behavioural problems among young people are the Child Behavior Checklist (CBCL), the Pediatric Symptoms Checklist (PSC), and the Strengths and Difficulties Questionnaire (SDQ). The CBCL consists of 113 questions on specific problematic behaviours to be answered by the parents or surrogates of school-aged children between 6 and 18 years old. The items can be organized into three main scales (total problems, internalizing problems, and externalizing problems) and eight subscales (withdrawn, somatic complaints, anxious/depressed, social problems, thought problems, attention problems, delinquent behaviour, and aggressive behaviour) [9, 10]. The PSC consists of 35 questions about a range of emotional and behavioural problems reported by parents of children and adolescents between 4 and 18 years old [11, 12]. These items can be organized into three sub-scales: attention problems, internalizing problems, and externalizing problems. The SDQ consists of 25 items about a number of emotional and behavioural problems experienced by children aged between 4 and 16 years. It has five subscales [13]: emotional symptoms, conduct problems, hyperactivity/attention problems, peer problems, and pro-social behaviour. The first four sub-scales can be combined into a total difficulties sub-scale, while the last is considered the strengths sub-scale. It has three versions, to be answered, respectively, by parents/caregivers, by teachers, and by the subjects themselves if over 11 years old; however, the self-reported version has also been used in younger children (8–13 years old) with good psychometric results [14]. The SDQ offers several advantages over the other instruments: 1) it is a brief instrument, and thus less burdensome; 2) it provides information from three different sources using a similar item structure, allowing comparison of results and a more comprehensive overall assessment of children and adolescents’ mental status; and 3) it assess both strengths and difficulties at the same time.

Another important consideration when selecting an instrument is the availability of studies exploring its validity and reliability in a population of the target age. For instance, in Chile, there is a standardized version of the CBCL for children between 6 and 11 years old [15]. It is worth mentioning that this country-specific version is not based on the updated general version available today. A Chilean version of the new CBCL has been validated, but for a population of 1½ to 5-year-old children [16]. The PSC has also been validated in Chile, in a population of socioeconomically vulnerable students in the first grade of primary school (aged 6 years) [17]. The Chilean version of the PSC consists of 33 items and has been extensively used for school-aged pre-adolescent students (under 12 years old) [18–21]. However, validation studies for adolescents are lacking. Finally, the psychometric properties of the SDQ have been evaluated in Chile for the parent-answered version for children between 4 and 11 years old. Therefore, to the best of our knowledge, no studies have explored the construct validity, reliability, and degree of agreement between the SDQ self-report and the parental report for Chilean adolescents. Additionally, we know of no SDQ studies for adolescents from Spanish-speaking, Latin-American countries.

The psychometric properties of the SDQ self-report and parental formats have been well established for adolescents in several countries, such as the United Kingdom [22], Germany [23, 24], Netherlands [25], Nordic countries [26], Russia [27], Australia [28], Austria [29], India [27], Yemen [27, 30], Bangladesh [27, 31], China [32, 33], Brazil [27], and other European countries [34]. The only studies exploring the construct validity and reliability of the Spanish version of the young and parental formats of this instrument came from Spain [35, 36].

Studies have confirmed the five theoretical dimensions in the adolescent self-reported version and in the parental version of the questionnaire, using exploratory and confirmatory factor analyses [37, 38]. However, other studies have failed to replicate the originally postulated five-factor solution [39–42]. Additionally, they have proposed a three-factor solution [41], combining the conduct and hyperactivity/attention problems as an ‘externalizing’ dimension and the emotional and peer problems as an ‘internalizing’ dimension, while keeping the pro-social sub-scale as a separate factor [43]. Furthermore, despite evidence that the five-factor model fits well across gender and ethnic groups for young children [44], a study gathering information from five European countries found that the number of factors could be country-dependent in the case of adolescents [40], and a Norwegian study found that factor loadings were different between pre-adolescents and adolescents [45]. Regarding reliability, some studies have shown adequate internal consistency [46–49], while others have reported low values for some sub-scales, especially for conduct and peer problems [50].

For adolescents in Spanish-speaking countries in Latin America, we have found no studies exploring the construct validity of the SDQ by means of confirmatory factor analysis (CFA), using parental and self-reported data. As mentioned earlier, we found only two studies using the SDQ in Chile, one exploring the psychometric properties of the parental reports on children between the ages of 4 and 11 [51], and one presenting the results of comparing the scores between early-adolescent Aymara [an indigenous South American nation] and non-Aymara students, using the self-reported, parental, and teacher versions [52].

Therefore, we see a knowledge gap concerning the performance of the SDQ in Spanish-speaking, Latin-American countries, specifically, its construct validity and reliability for adolescent populations. The aims of the present study are: i) to evaluate competing models of the latent structure of the SDQ, using confirmatory factor analysis; ii) to explore the reliability of the resulting sub-scales having the best fit; iii) to compare the degree of agreement between adolescent self-reports and parental reports, and their respective explanatory power; iv) to provide normative data for the SDQ adolescent and parental versions.

## Methods

### Participants

For the purposes of this study, we used SDQ data from two separate studies in similar, school-aged populations. The first study (Study 1) is being conducted in a vulnerable urban population in San Felipe, a small city north of Santiago. This is an ongoing longitudinal study exploring the factors associated with the development of health-promoting behaviours in early adolescents. As part of the baseline assessment, we administered the SDQ to the students (10 to 15 years old) and the parents. Some preliminary results of the cross-sectional analysis have been published [53]. The second study (Study 2) gathered data from students 9 to 15 years old, aiming to test the validity of the SDQ and of the Chilean-adapted version of the Olweus Bully/Victim Questionnaire Revised. This preliminary validation study is part of a larger, ongoing research called ‘The KiVa antibullying program in primary schools in Chile, with and without the digital game component, a randomized controlled trial’ [54]. We decided to present here analytical results from both studies because Study 1 gathered information from low-income families, while Study 2 gathered information from low-, middle-, and high-income families, allowing the latter set of results to be more representative of the adolescent Chilean population as a whole.

### Procedure and ethics

**Fig 1:**
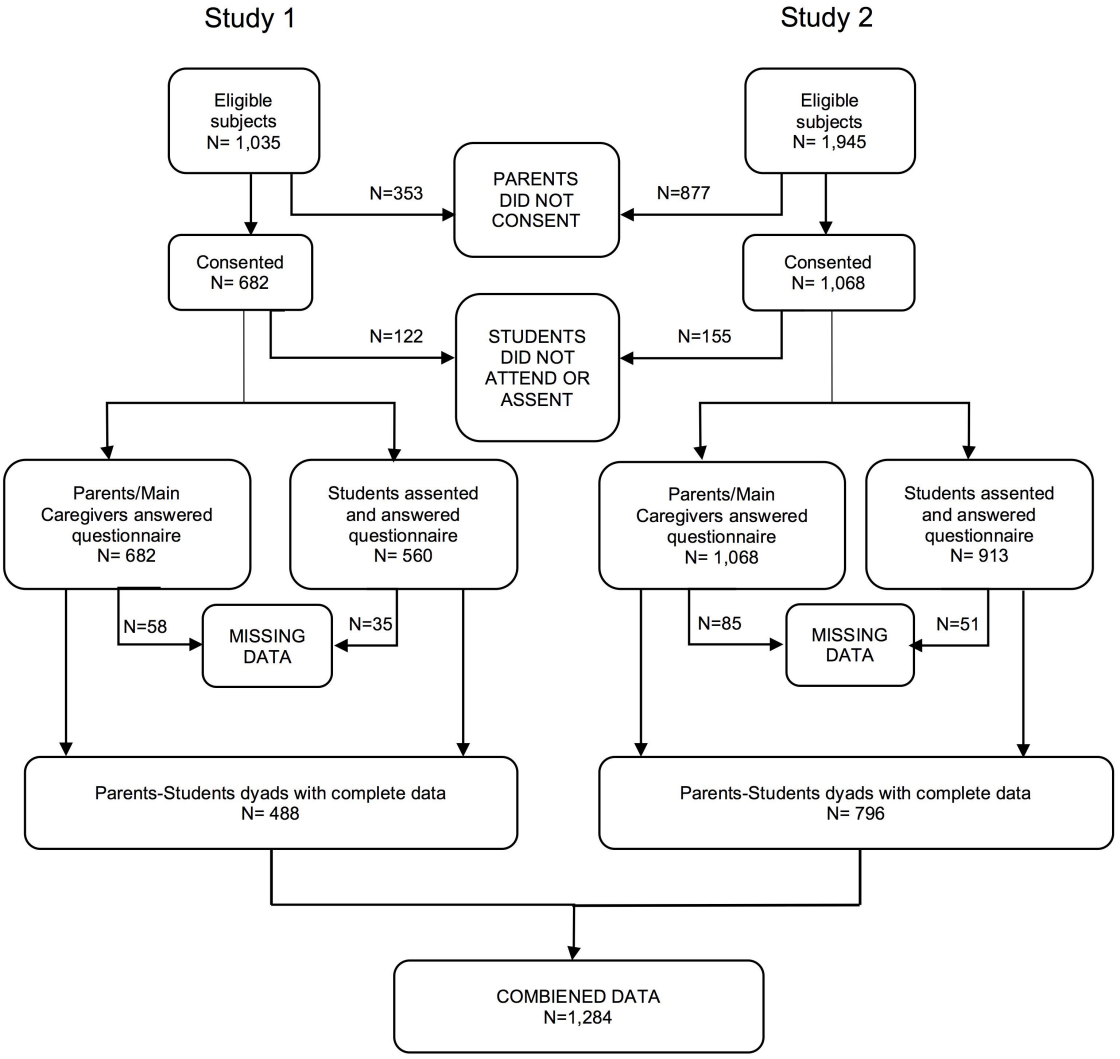
Flow chart.

In Study 1, we invited all urban, municipal, state-funded primary schools in San Felipe (n = 10) to participate after obtaining authorization from school board authorities. All ten schools agreed to participate. We informed the parents or main caregivers about the study and asked them to sign and informed consent to allow their children to participate. A total of 1,035 parents were contacted, 682 consented and answered the parental questionnaire. On the day of the assessment, 560 students assented to participate and answered the questionnaire (10 did not assent, and 112 were absent that day). A total of 488 parent-child dyads provided complete data. In Study 2, we invited five schools to participate. A total of 1,945 parents or main caregivers were contacted, and 1,068 consented and answered the parent version of the SDQ questionnaire. On the day of the assessment, 913 students assented to participate and answered the questionnaire (50 did not assent, and 105 were absent that day). A total of 796 parent-child dyads provided complete data. See Fig 1: Flow chart. In both studies, the parental SDQ questionnaire was answered by the main caregiver, which most of the time was the mother. Other main caregivers were the father or other significant family member such as grandmother.

Both studies had been approved by the Ethics Committee of the Universidad de los Andes. Written informed consent was obtained from parents and written informed assent from adolescents. The study posed no risks.

### Measures

#### Socio-demographic variables

Sex (0 = male; 1 = female), age (years) and socio-economic status (0 = low-income families; 1 = middle-income; 2 = high-income families) were collected. The socioeconomic status was based on the criteria of the 2009 National System for the Measurement of Education Quality, which gathers information from parents or main caregivers about their household income, and it was collected from the Ministry of Education.

#### Strengths and Difficulties Questionnaire (SDQ) [55–57]

This questionnaire has 25 items, divided into five subscales: emotional symptoms, conduct problems, hyperactivity-inattention problems, peer problems, and pro-social behaviour. These five subscales can be organized into two major sub-scales: strengths (pro-social behaviour) and difficulties (the other four subscales). Each item uses a three-point ordinal format to be answered with one of the following: 0 = not true; 1 = somewhat true; and 2 = certainly true. Five of the items are negatively worded in the original (i.e. is obedient, thinks before acting, has good attention, has good friends, is generally liked). Therefore, for compatibility in combining subscales into major subscales, their scores were reversed. The mean score for each subscale was then calculated (range 0–10). All scores for the difficulties subscales were added up to a total difficulties score (range: 0–40). The scores on the pro-social subscale were analysed independently (range: 0–10). The SDQ has been translated into more than 50 languages [10]. We used the authorized Spanish version and the scoring algorithms proposed by its author (for more information see: sdqinfo.com).

### Data analysis

For the purposes of this article, only responders with valid answers on all 25 items were included in the analyses.

Firstly, we summarized the socio-demographic variables and basic psychometric characteristics of the items using descriptive statistics, including means, standard deviations (SD), and, when necessary, frequencies and percentages. We used a structural equation modelling approach to CFA, to assess the structure of the proposed five-factor and three-factor models for the SDQ, in self-reports as well as in parental reports. Multivariate Mardia’s coefficients [58] and polychoric matrices were calculated to evaluate the distribution of the items. We ensured the adequacy of the matrices by assessment of the determinant, by the KMO index, and by Barlett’s test [59]. We also calculated the internal consistency of each factor by using McDonald’s Omega (ω), which can be interpreted as the square of the correlation between the scale score and the latent variable common to all the indicators [60]. The Omega index assumes a congeneric model, which means that factor loadings are allowed to vary, and it also takes into account the item-specific measurement error. Thus, it provides a more realistic estimate of true reliability than classical Cronbach’s Alpha values, being that both can be interpreted using the same threshold cut-off points.

We used the unweighted least squares (ULS) method for factor extraction, in view of its robustness [61]. Specifically, The ULS method does not provide inferential estimations based on the χ^2^ distribution (and therefore does not provide p-values), but it does not require any distributional assumption; it is robust and usually converges because of its computational efficiency; it tends to provide less biased estimates of the true parameter values than other procedures; and it shows good performance when working with polychoric matrices [62–65]. From a general perspective, we used we used the fit indices that the ULS reports such as the goodness-of-fit index (GFI), the adjusted goodness-of-fit index (AGFI), the normed-fit index (NFI), and the root-mean-square of the standardized residuals (RSMR). GFI and AGFI refer to the explained variance of the model, and values >0.90 are considered acceptable [66]. The NFI measures the proportional reduction in the adjustment function when going from null to the proposed model and is considered acceptable when >0.90 [67]. The RSMR is the standardized difference between the observed and the predicted covariance, indicating a good fit for values <0.08 [68]. From an analytical perspective, standardized saturations and the explained variance were considered. We also used a CFA approach to MTMM analysis [69]. This approach permits separation of the true variance on the constructs from variance resulting from measurement methods (self-report vs. parental report). The logic is that self-report and parental measures of the same construct should be highly correlated, but that measures of different constructs should have low correlations. We calculated squared factor loadings to estimate the explained variance in the sub-scales resulting from the underlying trait and the reporting method. The unexplained variance was termed uniqueness. We performed the same analyses according to age (≤11 vs >11) and socioeconomic status (Low vs Middle/High income) to assess potential differences.

We calculated the 25^th^, 50^th^, 75^th^, and 90^th^ percentile scores for each generated sub-scale, for the total sample and for each sex, for both the adolescent and the parental SDQ versions. We also present the normative data for age groups ≤11 and >11 years old.

All analyses were performed using STATA-14, SPSS-19, and Amos-7.

## Results

The materials used to produce the following results will be available upon request, including a detailed list of documents and all the data files needed in order for replication, as well as every step and the specific sequence the interested researchers should take into account to make data available [70]. Authors will post the referred materials in the group’s website, and/or will be send when asked for them [71].

### Characteristics of participants

Missing data from item responses varied from 0.2% to 1.0%. The final number of respondents in children and early adolescents (age range: 9–15 years) and their parents was 1,284 (54.0%, female). The mean age was 11.3 years (SD = 1.4). Students attended 4^th^- (13.6%), 5^th^- (26.2%), 6^th^- (24.1%), 7^th^- (24.9%), and 8^th^- (11.3%) year primary school. Of the student sample, 40.6% came from low-income families, 21.0% from middle-income families, and 38.4% from high-income families. Table 1 shows the general features of the whole sample, and divided by study.

**Table 1:** Descriptive features of parent-child dyads with complete data.

	Total sample % (95%CI) / Mean(SD) n = 1284	Study 1 % (95%CI) / Mean(SD) n = 488	Study 2 % (95%CI) / Mean(SD) n = 796
**Sex**			
**Girls**	46.0 (43.3–48.8)	50.4 (46.0–54.8)	56.2 (52.7–59.6)
**Boys**	54.0 (51.2–57.0)	49.6 (45.2–54.0)	43.8 (40.4–47.3)
**Age**	11.3 (1.4)	11.5 (1.2)	11.2 (1.5)
**Grade**			
**Year 4**	13.6 (11.8–15.5)	-	21.9 (19.1–24.9)
**Year 5**	26.2 (23.8–28.6)	34.2 (30.1–38.6)	21.2 (18.5–24.2)
**Year 6**	24.1 (21.8–26.5)	32.8 (28.8–37.1)	18.7 (16.2–21.6)
**Year 7**	24.9 (22.6–27.4)	33.0 (28.9–37.3)	20.0 (17.3–22.9)
**Year 8**	11.3 (9.7–13.1)	-	18.2 (15.7–21.1)
**Socioeconomic status**			
**Low**	40.6 (37.9–43.3)	100	4.1 (3.0–5.8)
**Medium**	21.0 (18.9–23.3)		33.9 (30.7–37.3)
**High**	38.4 (35.8–41.1)		61.9 (58.5–65.3)

### Psychometrics and construct validity of the SDQ

The polychoric matrix of the SDQ items using self-report data had a determinant of 0.008, the KMO test had a value of 0.87, Bartlett´s test gave 6,167.30 (df = 300; p < 0.001), and Mardia’s statistic was 48.24 (p < 0.001). In contrast, the matrix of the parental-report SDQ items had a determinant of 0.001, a KMO value of 0.87, a Bartlett´s test of 8,340.10 (df = 300; p < 0.001), and a Mardia’s statistic of 82.07 (p < 0.001).

Table 2 shows that the CFA fit indices for the SDQ were within acceptable values only in the case of the five-factor model ‒which in fact was originally proposed from a theoretical point of view‒ and they were better than in the case of the three-factor model, being that both the corresponding self-report and parental-report indices were adequate in the former.

**Table 2:** Confirmatory factor analysis of the SDQ.

Models	CMIN	NPAR	GFI	AGFI	NFI	RMSR
*Three factors*						
Self-report	594.79	53	0.924	0.910	0.875	0.092
Parental	554.35	53	0.932	0.918	0.900	0.101
*Five factors*						
Self-report	451.05	60	0.943	0.930	0.905	0.081
Parental	387.06	60	0.952	0.941	0.930	0.084
MTMM	704.06	41	0.997	0.988	0.993	0.026

Note: CMIN, Chi-square statistic, comparing the tested model and the independence model to the saturated model; NPAR, number of parameters in the model; GFI, goodness-of-fit index; AGFI, adjusted goodness-of-fit index; NFI, normed-fit index; RMSR, root mean square of the standardized residuals; MTMM, multitrait-multimethod.

**Fig 2:**
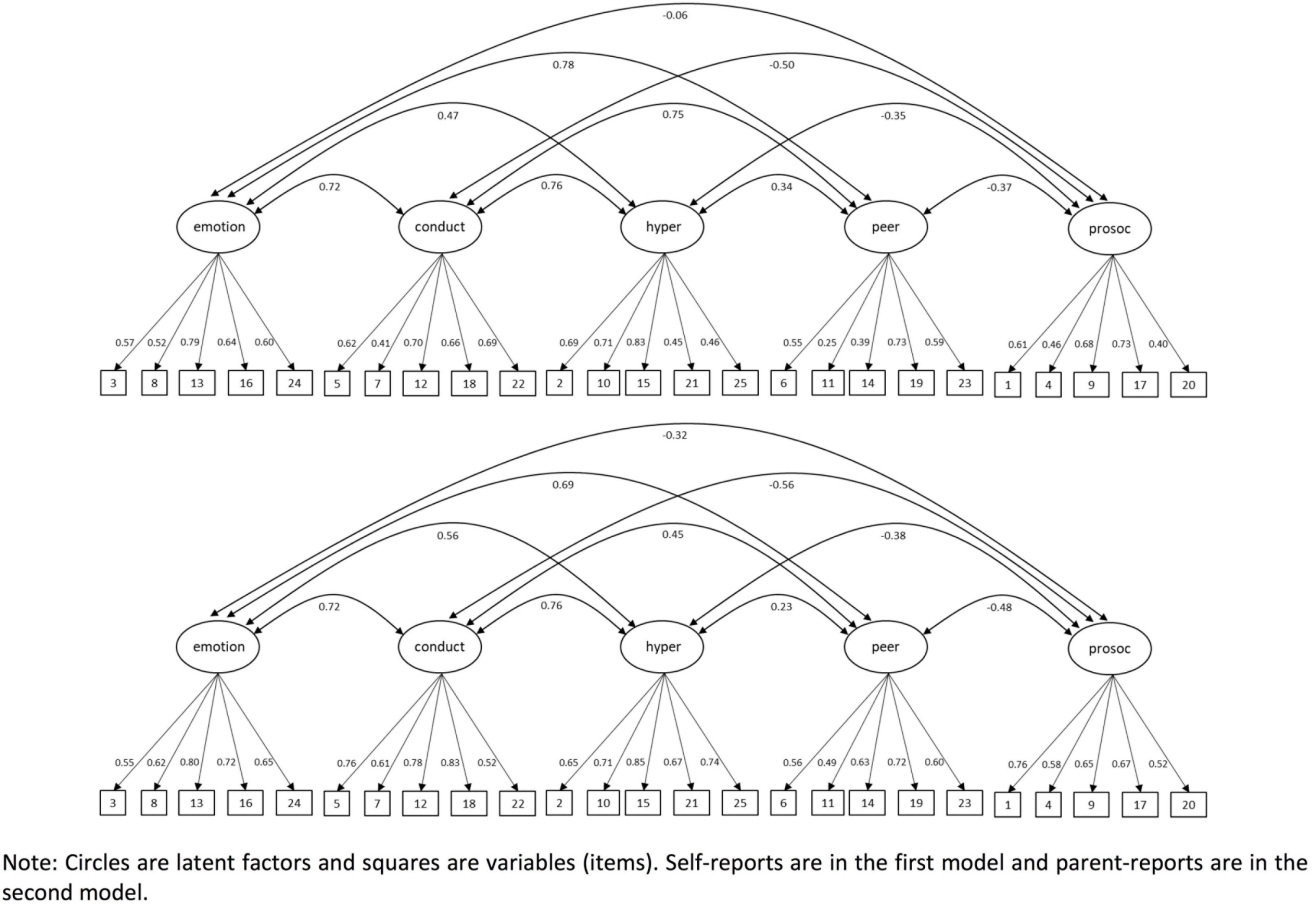
CFA parameters of the SDQ for self- and parent- reports (n = 1,284).

Table 3 shows the descriptive statistics and McDonald’s Omega values for the SDQ items and factors, and Fig 2 the weights and correlations between factors for the CFA both for self-report and parental-report scores. As we can see, the values and variability of the ‘steal’ item are low in self-report scores (mean = 0.20, SD = 0.50), but especially so in parental-report scores (mean = 0.04, SD = 0.26). The factorial loadings are adequate, although the ‘good friend’ item is a low outlier in the case of self-report scores (w = 0.25). In terms of reliability, parental reports are more consistent than self-reports. McDonald’s Omega values for self-reports range from 0.65 (‘peer problems’) to 0.77 (‘hyperactivity’) and, in the case of parental reports, from 0.76 (‘peer problems’) to 0.85 (‘conduct problems’, and ‘hyperactivity’).

**Table 3:** Descriptive and standardized factor loadings for SDQ items.

	Self-report			Parental-report		
	ω	Mean	SD	ω	Mean	SD
**Emotional symptoms**	**0.76**	**3.48**	**2.40**	**0.81**	**2.86**	**2.37**
Somatic (3)		0.57	0.71		0.56	0.70
Worries (8)		0.90	0.75		0.52	0.66
Unhappy (13)		0.51	0.68		0.38	0.63
Clingy (16)		0.93	0.77		0.77	0.74
Fears (24)		0.58	0.71		0.63	0.72
**Conduct problems**	**0.76**	**2.21**	**1.92**	**0.85**	**1.98**	**1.85**
Tempers (5)		0.58	0.71		0.80	0.74
Obedient (7)		0.70	0.59		0.57	0.61
Fights (12)		0.33	0.59		0.25	0.52
Lies (18)		0.40	0.63		0.32	0.52
Steals (22)		0.20	0.50		0.04	0.26
**Hyperactivity**	**0.77**	**3.91**	**2.39**	**0.85**	**3.69**	**2.56**
Restless (2)		0.79	0.75		0.62	0.72
Fidgety (10)		0.78	0.76		0.61	0.72
Distractible (15)		0.92	0.75		0.93	0.77
Thinks before acting (21)		0.69	0.63		0.77	0.61
Good attention (25)		0.74	0.62		0.75	0.68
**Peer problems**	**0.65**	**2.30**	**1.93**	**0.76**	**1.95**	**1.84**
Solitary (6)		0.40	0.65		0.46	0.68
Good friend (11)		0.31	0.63		0.20	0.46
Generally liked (14)		0.51	0.60		0.31	0.52
Bullied (19)		0.42	0.65		0.33	0.55
Adults (23)		0.66	0.74		0.65	0.70
**Pro-social behaviour**	**0.72**	**7.73**	**1.80**	**0.77**	**8.42**	**1.64**
Considerate (1)		1.61	0.55		1.74	0.48
Shares (4)		1.51	0.60		1.68	0.53
Caring (9)		1.55	0.56		1.70	0.51
Kind to kids (17)		1.71	0.53		1.81	0.46
Often volunteers to help (20)		1.36	0.64		1.50	0.59

Note: Values are means, standard deviations (SD), and McDonald’s Omega (ω).

In self-report scores, the correlations among the five constructs are strongest between ‘emotional symptoms’ and ‘peer problems’ (*r* = 0.78), while in parental reports, the strongest correlations are between ‘conduct problems’ and ‘hyperactivity’ (*r* = 0.76). The lowest correlations in self-reports are between ‘pro-social behaviour’ and ‘emotional symptoms’ (*r* = -0.06), while the lowest correlations in parental reports are between ‘hyperactivity’ and ‘peer problems’ (*r* = 0.23). See Fig 2.

### Trait and method components in the MTMM approach

**Fig 3:**
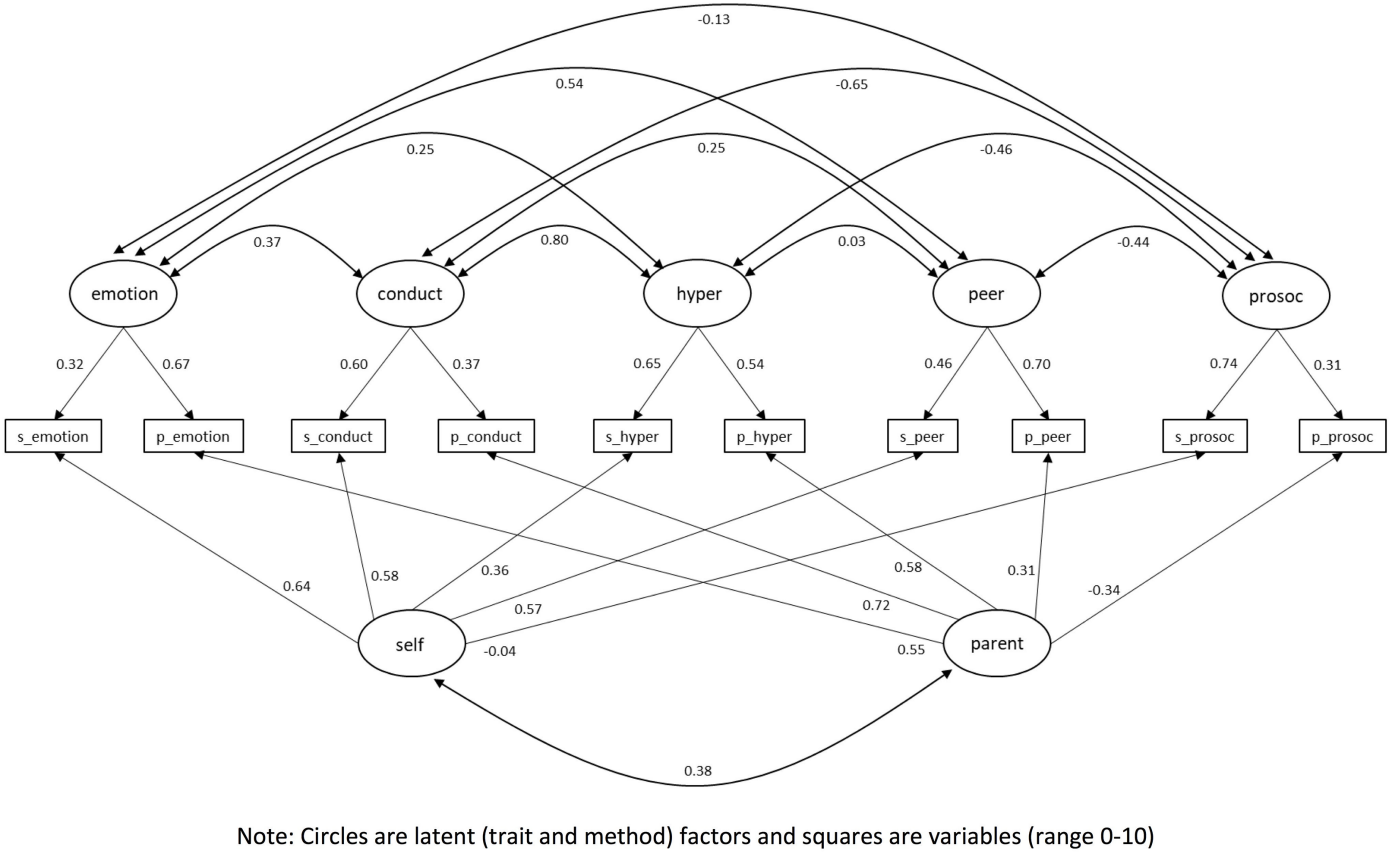
Structural MTMM model of the SDQ for Chilean adolescents (n = 1,284) using CFA.

The CFA approach to MTMM with two method factors and five trait factors has a very good fit to the model (Table 2). Fig 2 shows the CFA parameters for the MTMM approach, Fig 3 shows the structural parameters for the MTMM model, and Table 4 shows the trait and method variance components. As we can see, the trait variance components suggest that self-reports tend to be more discriminating on ‘pro-social behaviour’ (where they are practically unaffected by method) and ‘conduct problems’. On the other hand, parental reports seem to be particularly discriminating on ratings for ‘peer problems’ (where they are subject to low method effects) and ‘emotional symptoms’. The largest uniqueness value in self-reports is in ‘emotional symptoms’, while in parental reports it is in ‘pro-social behaviour’. Finally, we found moderately low correlations between the methods (*r* = 0.38).

**Table 4:** Trait and method variance components for the MTMM analysis.

	Measured variables	Trait	Method	Uniqueness
Self-report	Emotional symptoms	0.10	0.41	0.49
	Conduct problems	0.36	0.34	0.30
	Hyperactivity	0.42	0.13	0.45
	Peer problems	0.21	0.33	0.46
	Pro-social behaviour	0.55	0.01	0.44
Parental	Emotional symptoms	0.45	0.30	0.25
	Conduct problems	0.14	0.52	0.34
	Hyperactivity	0.29	0.34	0.37
	Peer problems	0.49	0.10	0.41
	Pro-social behaviour	0.10	0.12	0.78

We have produced additional results for CFA and MTMM analyses stratified by age (≤11 vs >11) and socioeconomic status (Low vs Middle/High income). These results are available in S1 File.

#### Normative data

Regarding the percentiles of the total difficulties scale, the values are similar between girls and boys, being lower for girls with a difference of one point. See Tables 5 and 6 for the total sample, Tables 7 and 8 for participants aged ≤11 years old, and Tables 9 and 10 for participants aged >11 years old. The percentiles in the self-reported SDQ are slightly lower than those in the parental SDQ.

**Table 5:** Normative data for total scores by sex based on adolescent self-report.

Percentiles	Emotional symptoms			Conduct problems			Hyperactivity-attentional problems			Peer problems			Total Difficulties scale			Pro-social Behaviour		
	Total	Girls	Boys	Total	Girls	Boys	Total	Girls	Boys	Total	Girls	Boys	Total	Girls	Boys	Total	Girls	Boys
**25**^**th**^	2	2	1	1	1	1	2	2	2	1	1	1	7	7	7	6	7	6
**50**^**th**^	3	4	3	2	1	2	4	4	4	2	2	2	11	11	11	8	8	7
**75**^**th**^	5	5	5	3	3	4	6	5	6	3	3	4	16	16	17	9	9	9
**90**^**th**^	7	7	7	5	4	5	7	7	7	5	5	5	21	21	21	10	10	10
**Total**	1284	693	591	1284	693	591	1284	693	591	1284	693	591	1284	693	591	1284	693	591

**Table 6:** Normative data for total scores by sex based on parental report.

Percentiles	Emotional symptoms			Conduct problems			Hyperactivity-attentional problems			Peer problems			Total Difficulties scale			Pro-social Behaviour		
	Total	Girls	Boys	Total	Girls	Boys	Total	Girls	Boys	Total	Girls	Boys	Total	Girls	Boys	Total	Girls	Boys
**25**^**th**^	1	1	1	1	1	1	2	1	2	0	0	1	5	5	6	7	8	7
**50**^**th**^	2	2	2	2	2	2	3	3	4	2	1	2	10	9	10	9	9	9
**75**^**th**^	4	4	4	3	3	3	5	5	6	3	3	3	15	14	15	10	10	10
**90**^**th**^	6	6	6	5	5	5	7	7	8	5	5	5	20	19	20	10	10	10
**Total**	1284	693	591	1284	693	591	1284	693	591	1284	693	591	1284	693	591	1284	693	591

**Table 7:** Normative data for total scores by sex based on adolescent self-report, age group 9–11.

Percentiles	Emotional symptoms			Conduct problems			Hyperactivity-attentional problems			Peer problems			Total Difficulties scale			Pro-social Behaviour		
	Total	Girls	Boys	Total	Girls	Boys	Total	Girls	Boys	Total	Girls	Boys	Total	Girls	Boys	Total	Girls	Boys
**25**^**th**^	1	1	1	1	1	1	2	2	2	1	1	1	7	7	7	7	7	6
**50**^**th**^	3	3	3	2	1	2	4	3	4	2	2	2	11	11	12	8	8	8
**75**^**th**^	5	5	5	3	3	4	5	5	6	4	4	4	16	15	17	9	10	9
**90**^**th**^	7	7	6.5	5	4	5	7	7	7	5	5	5.5	21	21	21	10	10	10
**Total**	709	379	330	709	379	330	709	379	330	709	379	330	709	379	330	709	379	330

**Table 8:** Normative data for total scores by sex based on parental report, age group 9–11.

Percentiles	Emotional symptoms			Conduct problems			Hyperactivity-attentional problems			Peer problems			Total Difficulties scale			Pro-social Behaviour		
	Total	Girls	Boys	Total	Girls	Boys	Total	Girls	Boys	Total	Girls	Boys	Total	Girls	Boys	Total	Girls	Boys
**25**^**th**^	1	1	1	1	1	1	2	1	2	0	0	0	5	5	6	8	8	7
**50**^**th**^	2	2	2	2	1	2	3	3	4	1	1	1	9	9	10	9	9	9
**75**^**th**^	4	4	4	3	3	3	5	5	6	3	2	3	14	14	14	10	10	10
**90**^**th**^	6	6	6	5	5	5	7	7	8	4	5	4	19	19	19	10	10	10
**Total**	709	379	330	709	379	330	709	379	330	709	379	330	709	379	330	709	379	330

**Table 9:** Normative data for total scores by sex based on adolescent self-report, age group 12–15.

Percentiles	Emotional symptoms			Conduct problems			Hyperactivity-attentional problems			Peer problems			Total Difficulties scale			Pro-social Behaviour		
	Total	Girls	Boys	Total	Girls	Boys	Total	Girls	Boys	Total	Girls	Boys	Total	Girls	Boys	Total	Girls	Boys
**25**^**th**^	2	2	1	1	1	1	2	2	2	1	1	1	7	7	7	6	7	6
**50**^**th**^	3	4	3	2	2	2	4	4	4	2	2	2	11	11	11	7	8	7
**75**^**th**^	5	6	5	3	3	4	6	6	6	3	3	3	16	16	16	9	9	9
**90**^**th**^	7	8	6	5	5	5	7	7	7	5	4	5	21	21	21	10	10	10
**Total**	575	314	261	575	314	261	575	314	261	575	314	261	575	314	261	575	314	261

**Table 10:** Normative data for total scores by sex based on parental report, age group 12–15.

Percentiles	Emotional symptoms			Conduct problems			Hyperactivity-attentional problems			Peer problems			Total Difficulties scale			Pro-social Behaviour		
	Total	Girls	Boys	Total	Girls	Boys	Total	Girls	Boys	Total	Girls	Boys	Total	Girls	Boys	Total	Girls	Boys
**25**^**th**^	1	1	1	1	0	1	2	1	2	1	0	1	6	5	7	7	7	7
**50**^**th**^	3	3	3	2	2	2	3	3	4	2	2	2	10	10	11	9	9	8
**75**^**th**^	5	5	5	3	3	4	5	5	6	4	3	4	15	15	16	10	10	9
**90**^**th**^	6	6	7	5	5	5	7	7	8	5	5	5	20	20	21	10	10	10
**Total**	575	314	261	575	314	261	575	314	261	575	314	261	575	314	261	575	314	261

## Discussion

This is the first study investigating the structure of the SDQ in a Spanish-speaking country in Latin America among adolescents and their parents. The results of our study support the originally proposed five-factor structure of the SDQ among early and middle adolescents and their parents/caregivers in Chile [13]. It appears to be a more plausible solution than the more recently proposed three-factor model [43]. However, we found high correlations between emotional symptoms and peer problems, and between conduct problems and hyperactivity, which may indicate latent, underlying internalizing and externalizing dimensions. Reliability values were in general adequate both for self-report and parent-report measures for all dimensions, although they were fair in the peer problems factor for self-report measures, being appropriate for parent-reports.

Exploring the structure of the SDQ stratified by age and socioeconomic status, the best fit was found in the parental report from middle/high households, and the worst fit was found in the self-report of students from low income households. The MTMM model was good in all strata. The correlation between self-report and parent report was similar among younger and older students, and among students coming from low income and middle/high households. Emotional problems were better explained by parental report among older students. While peer problems were better explained by parental report among students from middle/high households.

Some strengths of this study are the sample size, considering the challenge of collecting information from students and parents, and the representation of different socioeconomic backgrounds. Furthermore, normative data are provided, which may help future research to test cut-off points for determining the needs of adolescents with higher scores in difficulties sub-scales. This study, in line with most studies on SDQ, has been conducted in a population-based sample.

The factor loadings seem to be dissimilar between adolescents and parents. The factor loadings from the parents are higher than those from adolescents, which may indicate that adolescents experience their problems less distinctly than do their parents. We also notice that, in the case of adolescents, the lower, but still adequate, factor loadings were found in the negatively worded items. Furthermore, there was one item with a factor loading lower than de recommended cut-off point (<0.32) [72], namely ‘good friends’ (0.25). This methodological effect has been found in the SDQ previously [40, 73], and in other instruments [74, 75]. In our study, these results may be explained by the cognitive development of people at this age (9–15 years old), who may have had difficulty understanding the answer format or the direction of the intercalated questions. However, the results from parents all exceeded the recommended loading thresholds. Given the good factor structure, we recommend keeping these items for both adolescents and parents. However, in future research in Spanish-speaking countries, it will be important to report psychometric information bearing on whether to reconsider this recommendation, and to explore re-wording of the reverse items and assessing the effect on reliability.

Additionally, we found from the MTMM approach that the variance associated with the factor of methodological measure (self-report vs. parental report) was lower for hyperactivity problems (self-report), peer problems (parental report), and pro-social behaviour (both versions). However, for the remaining dimensions, the methodological component of the variance was high, suggesting the importance of having multi-informants when assessing psychopathology among adolescents. For example, when we see that the loading weight of the item ‘steals’ has half the weight in the parental report that it does in the self-report, we suspect that parents underestimate this symptom. This phenomenon is also found for other items such as ‘lies’ and ‘fights’, where adolescents may provide a better description of what they are doing than do parents.

This study has several limitations. Firstly, we did not have access to the entire range of ages addressed by the SDQ; therefore, our results are limited to the population studied here. Even though we have collected information from a large group of students and their main caregivers, there were many absent students the day of the survey, especially among low-income schools. Additionally, we could not access teacher reports to obtain a fuller picture of student behaviours. Several studies have shown the importance of having several informants to investigate students’ behaviours [76]. Even though our findings support a five-factor structure for the SDQ, it is possible that this instrument requires inversion of the wording of some of the items to improve understanding, especially among adolescents, which in turn may increase the reliability of some of the sub-scales. The usefulness of the normative data provided here is transitory; this data must be updated when we have better cut-off scores as a result of studies tapping both community and clinical populations. Therefore, next steps should be to explore diagnostic predictions made with the SDQ in Spanish-speaking countries in Latin America. Finally, the use of simple and short tools such as the SDQ may help to better investigate and understand the evolution of these symptoms during adolescence, and to explore mechanisms explaining the observed sex and cultural influences.

## Supporting information

S1 File Results for CFA and MTMM analyses stratified by age (≤11 vs >11) and socioeconomic status (Low vs Middle/High income). (PDF)
