# Supplementary material for: Mental health among children and adolescents: Construct validity, reliability, and parent-adolescent agreement on the ‘Strengths and Difficulties Questionnaire’ in Chile
Source: PLoS One. 2018 Feb 5;13(2):e0191809. doi: 10.1371/journal.pone.0191809 (PMC5798763; doi:10.1371/journal.pone.0191809)
Supplement: S1 File — (PDF) [file pone.0191809.s001.pdf]

S1

CFA of the SDQ according to age and socioeconomic status

Five factors model	CMIN	NPAR	GFI	AGFI	NFI	RMSR
**AGE**						
≤11 years old (n=709)						
Self-report	288.12	60	0.938	0.924	0.899	0.086
Parental	217.31	60	0.946	0.934	0.911	0.096
MTMM	435.81	41	0.997	0.987	0.992	0.029
>11 years old (n=575)						
Self-report	224.43	60	0.933	0.917	0.888	0.088
Parental	213.60	60	0.948	0.936	0.925	0.087
MTMM	6874.11	41	0.998	0.991	0.995	0.029
**SOCIOECONOMIC**						
Low status (n=521)						
Self-report	289.09	60	0.919	0.900	0.855	0.094
Parental	185.93	60	0.931	0.915	0.859	0.077
MTMM	10519.21	41	0.996	0.985	0.990	0.039
Middle/high status (n=763)						
Self-report	214.74	60	0.932	0.916	0.877	0.084
Parental	68.56	60	0.949	0.937	0.785	0.073
MTMM	4788.05	41	0.997	0.989	0.991	0.030

Note: CMIN, Chi-square statistic, comparing the tested model and the independence model to the saturated model; NPAR, number of parameters in the model; GFI, goodness-of-fit index; AGFI, adjusted goodness-of-fit index; NFI, normed-fit index; RMSR, root mean square of the standardized residuals; MTMM, multitrait-multimethod.

Structural MTMM model of the SDQ for ≤11 years old Chilean adolescents using CFA

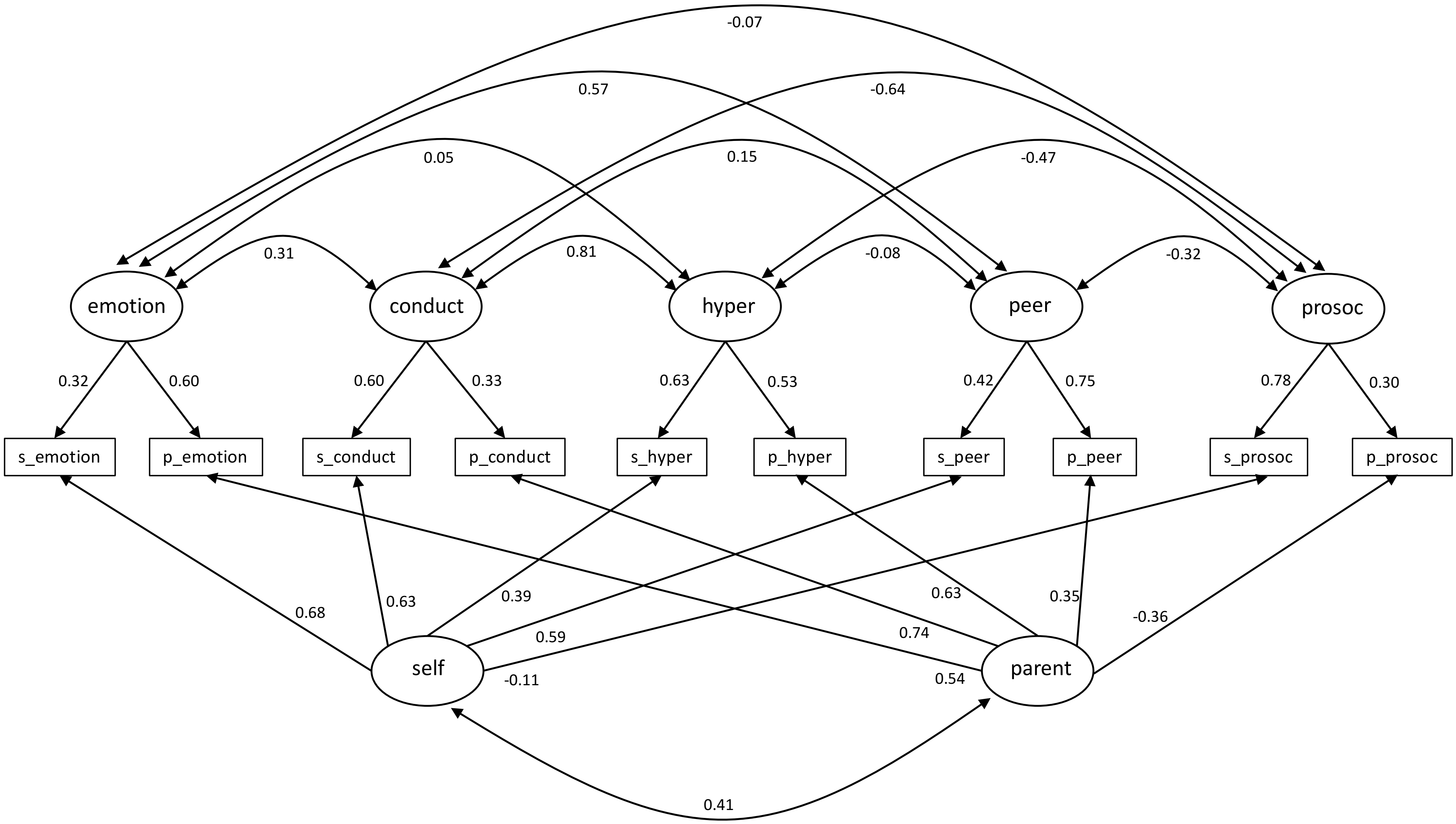

Note: Circles are latent (trait: emotional symptoms, conduct problems, hyperactivity-inattention problems, peer problems, pro-social behaviour; and method: self-reported, parent-reported) factors and squares are variables (as a sum of the corresponding items).

Trait and method variance components for ≤11 years old Chilean adolescents

	Measured variables	Trait	Method	Uniqueness
Self-report	Emotional symptoms	0.10	0.46	0.44
	Conduct problems	0.36	0.40	0.24
	Hyperactivity	0.40	0.15	0.45
	Peer problems	0.18	0.35	0.47
	Pro-social behaviour	0.61	0.01	0.38
Parental	Emotional symptoms	0.36	0.29	0.35
	Conduct problems	0.11	0.55	0.34
	Hyperactivity	0.28	0.40	0.32
	Peer problems	0.56	0.12	0.32
	Pro-social behaviour	0.09	0.13	0.78

Note: Trait: trait variance. Method: method variance. Uniqueness: unexplained variance.

Structural MTMM model of the SDQ for >11 years old Chilean adolescents using CFA

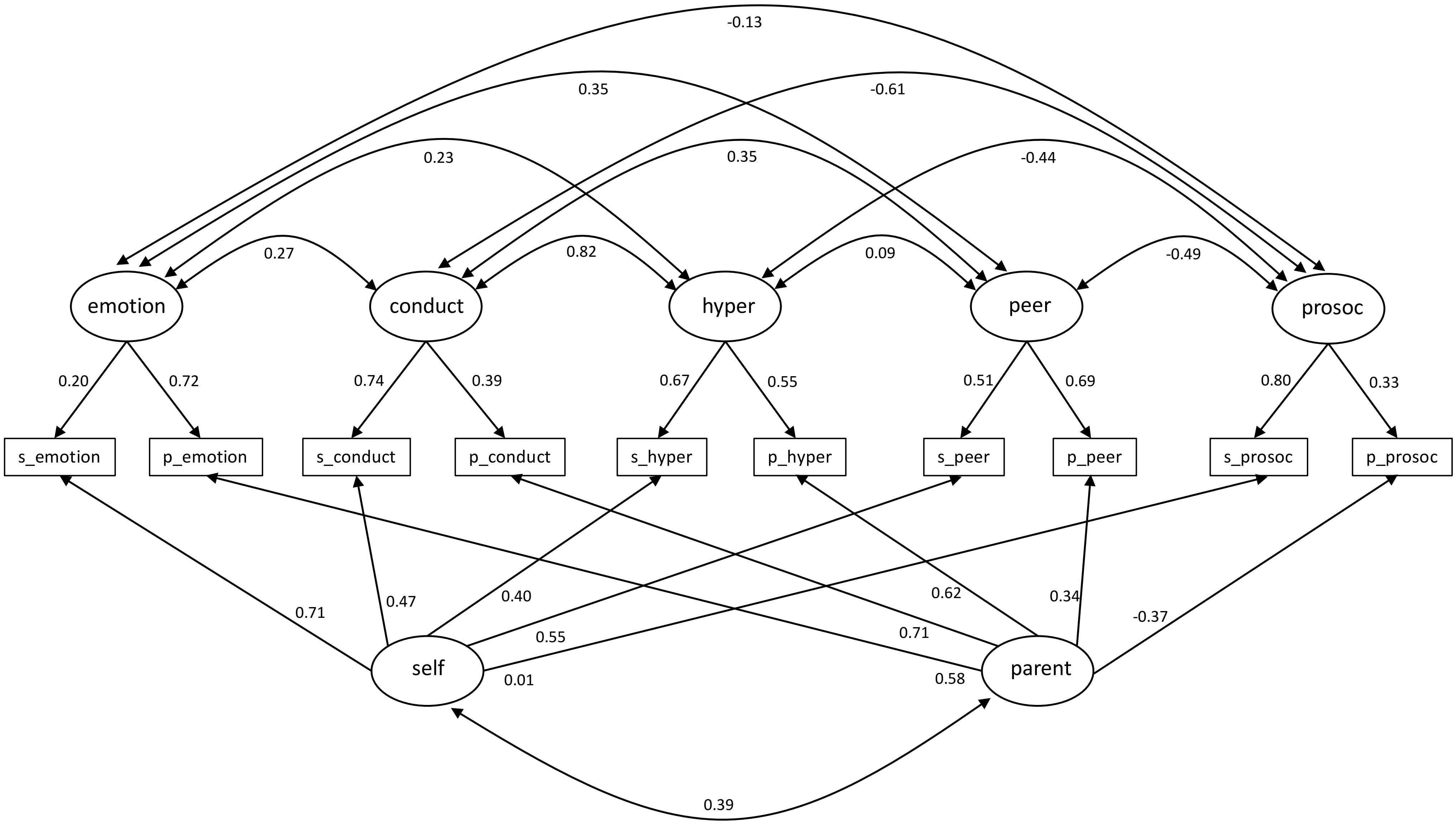

Note: Circles are latent (trait: emotional symptoms, conduct problems, hyperactivity-inattention problems, peer problems, pro-social behaviour; and method: self-reported, parent-reported) factors and squares are variables (as a sum of the corresponding items).

Trait and method variance components for >11 years old Chilean adolescents

	Measured variables	Trait	Method	Uniqueness
Self-report	Emotional symptoms	0.04	0.50	0.46
	Conduct problems	0.55	0.22	0.23
	Hyperactivity	0.45	0.16	0.39
	Peer problems	0.26	0.30	0.44
	Pro-social behaviour	0.64	<0.01	0.36
Parental	Emotional symptoms	0.52	0.34	0.14
	Conduct problems	0.15	0.50	0.35
	Hyperactivity	0.30	0.38	0.32
	Peer problems	0.48	0.12	0.40
	Pro-social behaviour	0.11	0.14	0.75

Note: Trait: trait variance. Method: method variance. Uniqueness: unexplained variance.

Structural MTMM model of the SDQ low socioeconomic Chilean adolescents using CFA

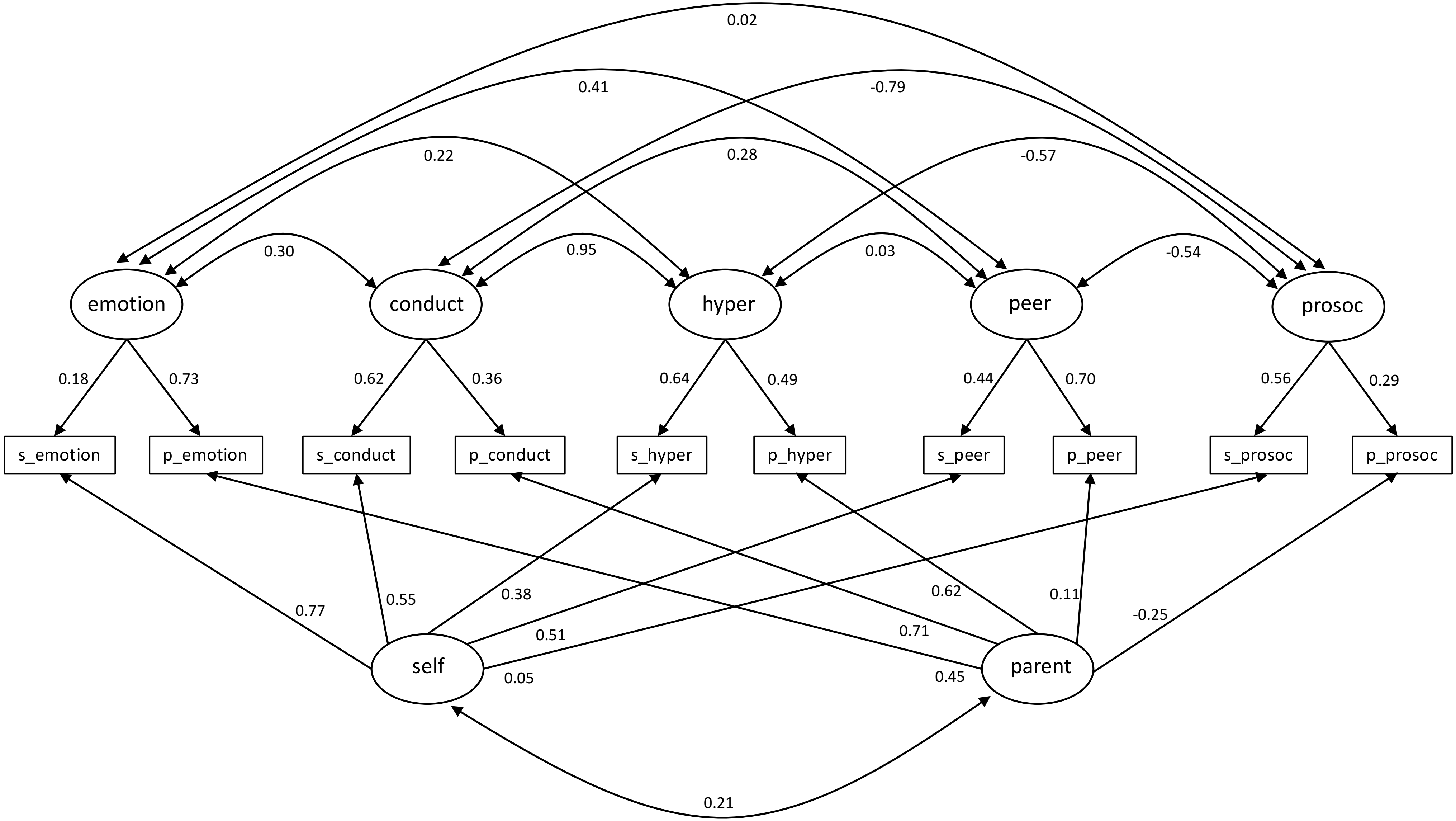

Note: Circles are latent (trait: emotional symptoms, conduct problems, hyperactivity-inattention problems, peer problems, pro-social behaviour; and method: self-reported, parent-reported) factors and squares are variables (as a sum of the corresponding items).

Trait and method variance components for low socioeconomic Chilean adolescents

	Measured variables	Trait	Method	Uniqueness
Self-report	Emotional symptoms	0.03	0.59	0.38
	Conduct problems	0.38	0.30	0.32
	Hyperactivity	0.41	0.14	0.45
	Peer problems	0.19	0.26	0.55
	Pro-social behaviour	0.31	<0.01	0.69
Parental	Emotional symptoms	0.53	0.20	0.27
	Conduct problems	0.13	0.50	0.37
	Hyperactivity	0.24	0.38	0.38
	Peer problems	0.49	0.01	0.50
	Pro-social behaviour	0.08	0.06	0.86

Note: Trait: trait variance. Method: method variance. Uniqueness: unexplained variance.

Structural MTMM model of the SDQ middle/high socioeconomic Chilean adolescents using CFA

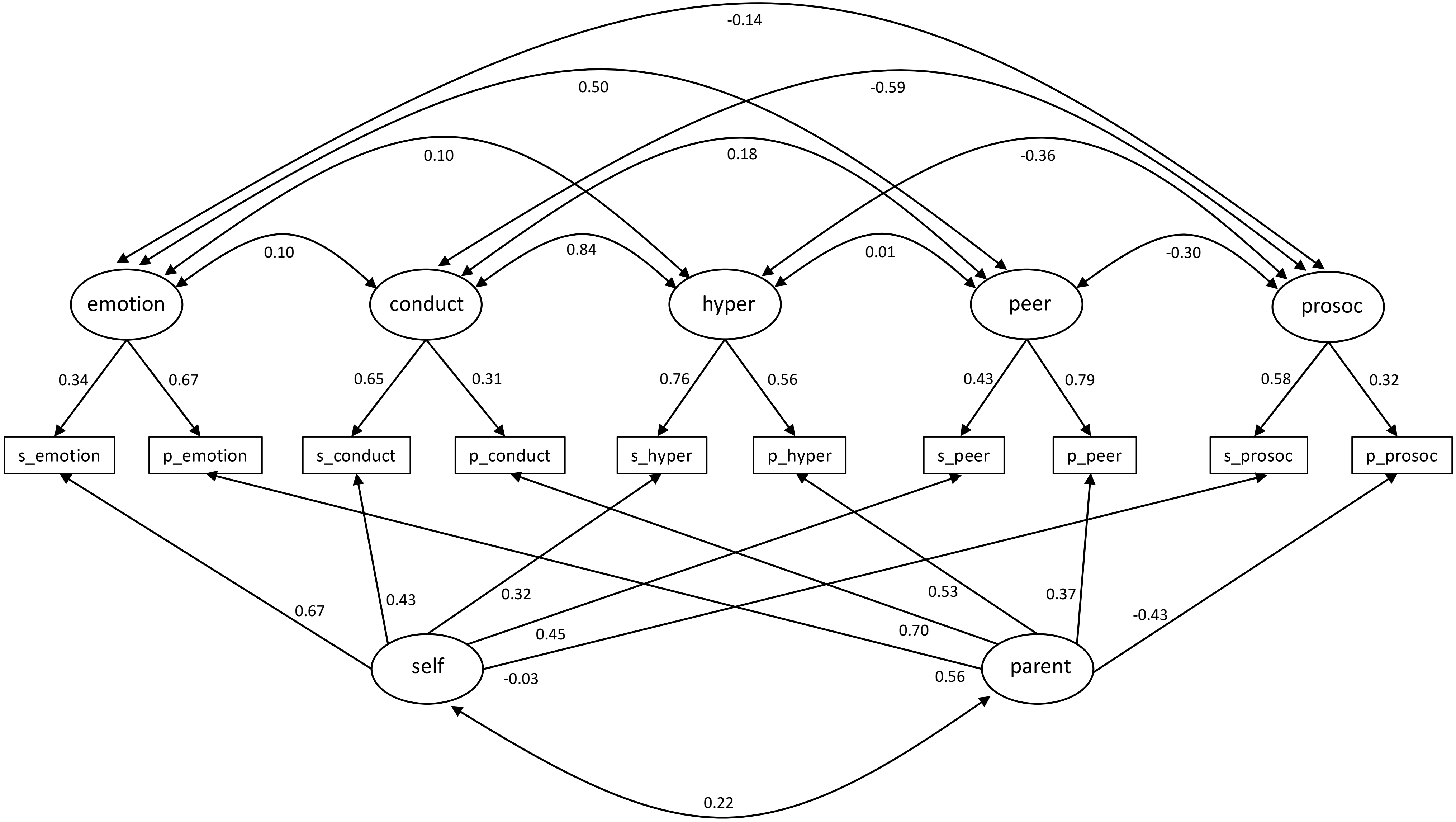

Note: Circles are latent (trait: emotional symptoms, conduct problems, hyperactivity-inattention problems, peer problems, pro-social behaviour; and method: self-reported, parent-reported) factors and squares are variables (as a sum of the corresponding items).

Trait and method variance components for middle/high socioeconomic Chilean adolescents

	Measured variables	Trait	Method	Uniqueness
Self-report	Emotional symptoms	0.12	0.45	0.43
	Conduct problems	0.42	0.19	0.39
	Hyperactivity	0.58	0.10	0.32
	Peer problems	0.19	0.20	0.61
	Pro-social behaviour	0.34	<0.01	0.66
Parental	Emotional symptoms	0.45	0.31	0.24
	Conduct problems	0.10	0.49	0.41
	Hyperactivity	0.31	0.28	0.41
	Peer problems	0.62	0.14	0.24
	Pro-social behaviour	0.10	0.19	0.71

Note: Trait: trait variance. Method: method variance. Uniqueness: unexplained variance.
